# Body Mass Index and Gonadotropin Hormones (LH & FSH) Associate With Clinical Symptoms Among Women With Polycystic Ovary Syndrome

**DOI:** 10.5539/gjhs.v7n2p101

**Published:** 2014-09-28

**Authors:** Seddigheh Esmaeilzadeh, Maryam Ghanbari Andarieh, Reza Ghadimi, Mouloud Agajani Delavar

**Affiliations:** 1 Fatemezahra Infertility and Reproductive Health Research Center, Department of Obstetrics and Gynecology, Babol University of Medical Sciences, Babol, Iran; 2 Fatemezahra Infertility and Reproductive Health Research Center, Babol University of Medical Science, Babol, Iran; 3 Fatemezahra Infertility and Reproductive Health Research Center, Department of Social Medicine and Health, Babol University of Medical Sciences, Babol, Iran; 4 Fatemezahra Infertility and Reproductive Health Research Center, Department of Midwifery, Babol University of Medical Sciences, Babol, Iran

**Keywords:** BMI, polycystic ovaries, polycystic ovary syndrome

## Abstract

To evaluate the relevance of body mass index (BMI), luteinizing hormone (LH), follicle-stimulating hormone (FSH) and LH/FSH ratio with clinical symptoms in polycystic ovary syndrome (PCOS) women.

**Methods:**

We reviewed the medical records of all women visited in the PCOS Clinic of Babol (Iran) from 2008 to 2012. A retrospective cross-sectional study was conducted on 175 PCOS women; aged 18–38 years diagnosed based on the Rotterdam criteria. Among the PCOs women, the prevalence of oligomenorrhea, acne, and hirsutism were found to be 92.0%, 31.4%, and 78.9%, respectively. Positive finding of polycystic ovaries was observed in 89.1% of PCOS women with by using sonography. A total of 69.2% overweight/obesity patients had polycystic ovary morphology on ultrasound image. Compared with non- overweight/obesity, the adjusted OR of PCOS women for sonographic view of polycystic ovaries was 4.33 (95% CI, 1.42-13.15, p=0.001), Nevertheless, the adjusted odds ratio (OR) showed no significant associations between LH, FSH, and LH/FSH ratio with clinical symptoms in these women. The findings of this study indicated that the overweight/obese women with PCOS are at an increased risk for sonographic view of polycystic ovaries. Therefore, it is suggested that successful weight loss is the most effective method of restoring ovulation, menstruation that should be used as major advice in obese PCOS patients.

## 1. Introduction

Polycystic ovary syndrome (PCOS) is best defined as the most frequent endocrine problem in women of reproductive age, which increase the risk of infertility. It includes the clinical sign such as oligo or anovulation, hirsutism, acne, and polycystic ovaries on ultrasound, and affected an estimated in 12–21% of women of reproductive age. The diagnosis is base on Rotterdam consensus criteria even when the syndrome is associated with a wide range of symptoms. Up to 70% of PCOS women stay undiagnosed. Moreover, PCOS patients may show LH elevated, LH/FSH ratio increased (>2), with FSH normal; however, this is not part of the diagnostic criteria. Women with PCOS are at risk for weight gain (Teede, Deeks, & Moran, 2010; Boyle, & Teede, 2012; Legroet al., 2013; March et al., 2010). Obesity is a common finding of women with PCOS, but it is not part of the diagnostic criteria. The national guideline highlights the key role of obesity in PCOS. Studies consistently show a higher prevalence of PCOS in women who are overweight and obese, and up to 30% of indigenous women who had a body mass index (BMI) >30 kg/m^2^ met PCOS diagnostic criteria. PCOS requires a multidisciplinary team to manage. Meanwhile there is still controversy over essential diagnostic investigations in PCOS. LH/FSH ratio greater than two has considered as “gold standard” in PCOS diagnosis for a long time. It is an intriguing problem to recognize the role of LH and furthermore to evaluate the usefulness of gonadotropin ratio in PCOS diagnosis (Teede, Deeks, & Moran, 2010; Boyle, Cunningham, O’Dea, Dunbar, & Norman, 2012; Diamanti-Kandarakis, Kandarakis, & Legro 2006). A study found 38% of their PCOS cases was overweight (Body Mass Index >25 kg/m^2^). The obesity was significantly associated with an increased risk of hirsutism, menstrual cycle disturbance. Furthermore, loss of body weight-induced by diet-has important effect, since it reduces blood androgen levels and can improve ovulation (Moran et al., 2013). Preliminary observations suggest that a gradual normalization of menstrual cycle abnormalities occurs in PCOS patients with increasing FSH and LH levels. In contrast, other investigators could not confirm such changes. In spite of the extensive study of PCOS, little information is available concerning the potential effect of body weight and hyperandrogenaemia especially on the clinical symptoms such as acne, hirsutism and oligomenorrhea. The objective of the present study was to evaluate the mutual relation of increasing weight and gonadotropin levels on clinical symptoms in PCOS women.

### 2. Methods and Materials

The ethics committee of Babol University of Medical Science approved this study. In this study, we reviewed the medical records of all women seen in Babol University of Medical Sciences PCOS clinic from 2008 to 2012 in Babol, Iran. In the clinic, all the guidelines of Rotterdam ESHRE/ASRM Consensus were followed for the diagnosis of 225 women with PCOS were identified (Revised 2003 consensus on diagnostic criteria, 2012). However, we found just175 women met the inclusion criteria of aged 18-38 years. The patients having any major systemic illness, congenital adrenal hyperplasia, hyper prolactinemia, acromegaly, thyroid dysfunction, and Cushing’s syndrome, androgen secreting neoplasm, and other pituitary or adrenal disorders, alternate androgen-excess disorders and risk factors for endometrial cancer, mood disorders, obstructive sleep apnea, diabetes, and cardiovascular disease, functional hypothalamic amenorrhea based on clinical findings and laboratory investigations were excluded.

The BMI was calculated as weight (kg) /height squared (m^2^). As per the Standard Consensus Statement of body mass index (BMI) for Iranian population, patients were grouped as normal weight <25 and obese (≥ 25). The severity of hirsutism scored according to the Ferriman-Gallwey (FG) scoring system. A score of 7 to 9 FG considered as mild hirsutism, 10-14 moderate and 15 or more severe hirsutism (Panidis et al., 2010).

### 2.1 Statistical Analysis

Data were analyzed using SPSS statistical software version 18.0 for Windows. Descriptive statistics were used to describe characteristics of PCOS women. The difference association between BMI, LH, FSH and LH/FSH with oligomenoreh, acne and hirsutism and polycystic ovaries were done by using chi square. Multiple logistic regression was used to analyze association between study variables. The odds ratio (OR) were presented together with their 95% CI. All independent variables that met the above criteria were included in the multiple logistic regressions. Adjustments were made for independent variables: including age, BMI, LH, FSH, LH/FSH. Statistical significance was set at a P value less than .05.

## 3. Results

Table 1 shows information regarding the characteristics of PCOS women. The mean age and BMI of the women was 23.5±4.6 years 27.7±5.9 Kg/m^2^, respectively. Among the PCOs women, 55 women (31.0%) had acne, in addition, the majority, and 161 women (92.0%) had oligomenoreh. One hundred thirty and eight (78.9%) had hirsutism with Ferriman and Gallwey (FG) score ≥8.

**Table 1:** Characteristics of participants, women aged 18 to 38 years (n=175)

Variable	Mean	SD
Age (year)	23.5	4.6
Menarch age (year)	12.2	1.2
BMI (kg/m^2^)	27.7	5.9
Hirsutism Score	11.7	6.0
Bleeding in mens	1.8	1.0
FSH	6.6	2.0
LH	8.1	8.5
LH/FSH	1.4	1.5
	N	%
Oligomenoreh	161	92.0
Acne	55	31.4
Hirsutism	138	78.9
Positive finding of polycystic ovaries in sonography	156	89.1

BMI = body mass index; FSH = follicle stimulating hormone; LH = luteinizing hormone.

As shown in Table 2, chi-square test revealed statistically significant difference in overweight/obesity and polycystic ovaries. There was no statistically significant difference in LH, FSH, and LH/FSH ratio with oligomenoreh, acne, hirsutism and sonographic view of polycystic ovaries.

**Table 2:** Body mass index and gonadotropins of PCOS women according to occurrence of clinical symptoms among women with polycystic ovary syndrome

Variable	LH/FSH>2/1 N (%)	FSH>=7 N (%)	LH>=10 N (%)	BMI>=25 N (%)
Oligomenoreh	108(67.1)	34(21.1)	63(39.1)	25(15.5)
Acne	33(60.0)	12(21.8)	23(41.8)	6(10.9)
Hirsutism	92(66.7)	30(21.7)	53(38.4)	25(18.1)
Polycystic ovaries	108(69.2)*	32(20.5)	60(38.5)	24(15.4)

* P value <0.05 (P-values obtained from chi-square tests).

Table 3 presented the adjusted odds ratio for BMI and gonadotropin hormones of PCOS women for association oligomenoreh, acne, hirsutism, and polycystic ovaries. Compared with non-overweight/obesity, the adjusted OR of PCOS women for sonographic view of polycystic ovaries was 4.33(95% CI, 1.42-13.15, p=0.001). However, we could not find considerable relationship between BMI and the other clinical symptoms. Inaadition, no significant association was found between gonadotropins and polycystic ovary syndrome (PCOS) women of PCOS.

**Table 3:** Adjusted* odds ratios (OR) for body mass index and gonadotropins of PCOS women according to occurrence of clinical symptoms among women with polycystic ovary syndrome (n=175)

Variable		LH/FSH>2/1 N (%)	FSH>=7 N (%)	LH>=10 N (%)	BMI>=25 N (%)
Oligomenorrea	161(92.0)	1.43(0.428-4.80)	3.06(0.46-20.45)	1.39(0.37-4.89)	0.43(0.08-2.38)
Acne	55(31.4)	0.64(0.31-1.34)	1.58(0.60-4.16)	1.08(0.55-2.13)	0.40(0.12-1.29)
Hirsutism	138(78.9)	0.74(0.32-1.72)	1.05(0.34-3.26)	0.83(0.39-1.80)	0.36(0.08-1.53)
polycystic ovaries	156(89.1)	4.33(1.42-13.15)**	1.65(0.39-7.01)	0.97(0.35-2.74)	0.61(0.14-2.71)

* Potential confounders used in all analyses were age, age at menarche, BMI, LH, FSH, and LH/FSH Ratio.

** P value <0.05 (P-values obtained from binary logistic regression).

## 4. Discussion

The finding of the present study indicated that the overweight/obese women with PCOS are at an increased risk for sonographic view of polycystic ovaries (Alnakash & Al-Tae’e, 2007). PCOS is reported to be more common in mean age of 23.5, proposing that due to a physiological decline of the follicular cohort leading to a normalized ovarian ultrasonographic appearance with advancing age. In Wijeyaratne et al. (2005) study, those with PCOS had significantly higher median BMI. Regarding BMI, 69.2% of our overweight/obese patients had polycystic ovary morphology. This result is higher than what is reported by Pasquali et al who showed that about 35% of the women with PCOS are overweight or obese or respectively (Pasquali, Vicennati, & Gambineri, 2007). Our higher incidence of overweight may be linked to the lack of exercise amongst women and fatty food habits adopted in this city. The importance of the issue demonstrated when Wijeyaratne concluded Increasing BMI was significantly related to an increasing trend in the proportion of women with the metabolic syndrome (Wijeyaratne et al., 2005).

Hirsutism is a good marker for hyperandrogenism especially when considering obesity. In the present study, hirsutism have shown in more than 78.9% of PCOS women and the rate of oligomenorrhea, acne, were found to be 92.0%, 31.4%. Wijeyaretne et al supported this finding. From Wijeyaratne’ PCOS patients, 74.6% had significant hirsutism, Acne was present in 39.2% patients. We may not agree with Kiddy et al. (1990) found there is increased frequency of hirsutism in obese compared with lean PCOS women. Whereas, all overweight /obese patients with and they had suffered from abnormally excessive hair growth.

Several studies supported that the investigators are not sure that higher BMI, LH, FSH and LH/FSH ratio necessarily indicate a greater incidence of PCOs features. For example, Sharquie KE concluded that, LH/FSH ratio has little effect in diagnosing polycystic ovarian syndrome. Dinka et al reported that no significant association was showed between the hirsutism grade, BMI and hormonal parameters examination (Sharquie, Al-Bayatti, Al-Ajeel, Al-Bahar, & Al-Nuaimy, 2007; Dinka, Lana, Zrinka, & Iva, 2013). We tried to find correlations between BMI, LH, FSH and LH/FSH ratio with PCOS manifestations (menstrual patterns, acne, sonographic view of polycystic ovaries, and hirsutism). Banaszewska et al reported abnormal LH/FSH ratio when it is greater than 2 and 4.5% of PCOS women having an elevated ratio. However they have shown that the mean LH/FSH ratio was not statistically significant difference between the PCOS and non PCO in his study (Banaszewska, Spaczynski, Pelesz, & Pawelczyk, 2003). Another investigation in Saudi also has been reported that regardless of the age and weight factors, Saudi patients with PCOS have higher levels of LH/FSH; but have lower levels of FSH compared to controls (Fakhoury et al., 2012). Kiddy et al. (1990) found an inverse correlation of FSH with BMI in obese PCOS. While Yanira et al revealed an inverse correlation between LH and BMI in PCOS (Yanira et al., 2006) but Insler et al determined a significantly higher level LH in non obese PCOS women compared with obese PCOS subjects (Insler et al., 1993). However, we found no significant associations between LH, FSH, and LH/FSH ratio with BMI of the PCOs women.

A positive finding of our research demonstrated that of all patients underwent ultrasonography, 89.1% showed PCOs. This amount is consistence with Wijeyaratne et al. (2005) that claimed 82.5% of women showed PCOs in ultrasonography. Our overweight/obese women had 4.33 adjusted OR compared with non- overweight/obesity PCOS women for sonographic view of polycystic ovaries. Although, some investigators like Dunaif et al resulted that for the diagnosis of the syndrome, polycystic morphology is not initially essential (Dunaif, 1997). But Adams et al. (1985) described that the most sensitive diagnostic marker for PCOS seen by vaginal ultrasound, even, Giuseppe Loverro claimed that ultrasound measurement of ovarian stroma may predict hyperandrogenism degree; prothrombotic factors and cardiovascular risk in patients with PCOS (Loverro et al., 2010) and Takahashi et al. (1995) reported that transvaginal ultrasound criteria of PCOS observed in 94% of PCOS women.

In conclusion, our study concluded that the overweight/obese women with PCOS are at an increased risk for sonographic view of polycystic ovaries. It has proved that age ≥35 years, BMI ≥25 kg/m^2^ and acne are as significant predictors of metabolic disorder in PCOS women. Therefore, it is suggested that successful weight loss is the most effective method of restoring ovulation and menstruation that should be used as major advice in obese PCOS patients.
